# Application of CCG Sensors to a High-Temperature Structure Subjected to Thermo-Mechanical Load

**DOI:** 10.3390/s16101686

**Published:** 2016-10-13

**Authors:** Weihua Xie, Songhe Meng, Hua Jin, Chong Du, Libin Wang, Tao Peng, Fabrizio Scarpa, Chenghai Xu

**Affiliations:** 1 Centre for Composite Materials and Structures, Harbin Institute of Technology, Harbin 150080, China; michael@hit.edu.cn (W.X.); wanglibin_hit@163.com (L.W.); boypengtao@163.com (T.P.); hit-xuchenghai@163.com (C.X.); 2 Shanghai Advanced Research Institute, Chinese Academy of Sciences, Shanghai 201210, China; duc@sari.ac.cn; 3 Advanced Composites Centre for Innovation and Science (ACCIS), University of Bristol, Bristol BS8 1TR, UK; f.scarpa@bristol.ac.uk

**Keywords:** fibre optic sensors, strain and temperature, chemical composition gratings (CCGs), high temperature application, hot structure, thermo-mechanical

## Abstract

This paper presents a simple methodology to perform a high temperature coupled thermo-mechanical test using ultra-high temperature ceramic material specimens (UHTCs), which are equipped with chemical composition gratings sensors (CCGs). The methodology also considers the presence of coupled loading within the response provided by the CCG sensors. The theoretical strain of the UHTCs specimens calculated with this technique shows a maximum relative error of 2.15% between the analytical and experimental data. To further verify the validity of the results from the tests, a Finite Element (FE) model has been developed to simulate the temperature, stress and strain fields within the UHTC structure equipped with the CCG. The results show that the compressive stress exceeds the material strength at the bonding area, and this originates a failure by fracture of the supporting structure in the hot environment. The results related to the strain fields show that the relative error with the experimental data decrease with an increase of temperature. The relative error is less than 15% when the temperature is higher than 200 °C, and only 6.71% at 695 °C.

## 1. Introduction

Over the past two decades many fibre Bragg grating (FBG)-based sensors have been developed for applications in high-temperature environments due to their unique features [1,2,3,4]. Some examples of the interesting characteristics that characterise FBGs are their low weight, compact size, electromagnetic and radiofrequency compliance, durability, long-distance transmission capabilities and good heat resistance [5,6]. FBG sensors are regrouped into classes that include type I, type II and long-period gratings, photonic crystal fibres, sapphire gratings and chemical composition gratings (CCGs), the latter also commonly called regenerated FBGs (RFBGs). RFBGs have been used in environments up to 1295 °C [7] and are typically produced from hydrogen-loaded optical fibres by applying an annealing treatment at temperatures between 800 °C and 1000 °C [8]. The original grating is completely erased and a new grating refractive index modulation is generated following the annealing process. The main advantages of RFBGs are their ultrahigh temperature stability, good grating qualities and their potential for measuring reflected light.

To produce stable thermal sensors for high temperature environments Fokine [9,10,11,12,13,14], Zhang [15,16], Canning [7,17,18,19], Barrera [20,21,22], Guan [23,24,25] and others [26,27,28,29,30] have devoted significant efforts to the study of the design, production and related technologies of RFBGs. Recently, a number of research groups have also begun to focus their efforts on the application of RFBGs in high-temperature environments. Issues related the utilisation of RFBGs in these challenging environmental conditions are related to the safe packaging and installation of the sensors in structures, because silica-based RFBGs are extremely brittle and fragile after the annealing process [1]. Méndez et al. [31,32], Selfridge et al. [33], Sai Prasad and co-workers [34] have used metal substrates to provide the connection between the optical fibre sensors and the structure. Mamidi et al. [35], Reddy et al. [36], Barrera et al. [37,38] and Azhari et al. [39] have also used a metal or ceramic tube to encapsulate the RFBGs to perform measurements in high temperature environments. 

In a previous work [40] we have described a simple method to apply CCG sensors on ultra-high temperature ceramic materials (UHTCs) in order to measure strains in thermal environments up to 1000 °C. This background work has shown the promise of using CCGs for applications in high temperature environments. Aerospace hot structures and thermal protection systems (TPS) are examples of some of the most likely applications for CCG sensors, which are subjected to simultaneous thermal and mechanical load [41]. The thermo-mechanical coupling makes the measurement of high temperature strains however extremely difficult and expensive. In this paper we present an experimental method that can identify the presence of a simultaneous thermal and mechanical load on a structure subjected to high temperatures by using CCGs. We carry out a thermo-mechanical experiment related to the application of CCGs on an ultra-high temperature ceramic material (UHTC) structure. The results from the experiments and from theoretical formulas are compared and analysed. We also develop a FE model to simulate the experiments and explain the failure mechanism based on the stress distribution and strain field variation. The relative errors between the CCG sensor and FE method related to the strain measurements are also compared and discussed.

## 2. Experimental Design 

**Figure 1:**
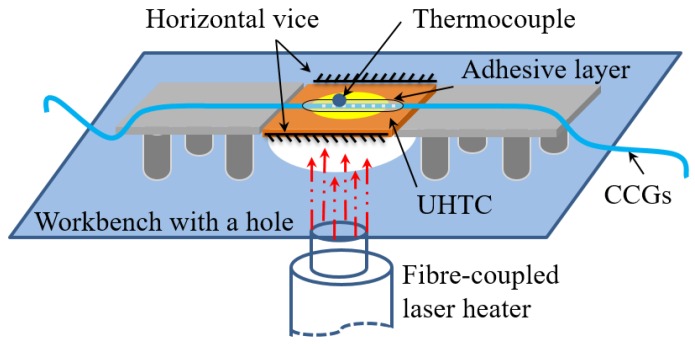
Scheme illustrating thermal-mechanical experimental apparatus.

The experimental setup used in this work (Figure 1) was designed to simulate a realistic service condition represented by a thermal protection shield (TPS) on a hot external structure, like in hypersonic vehicles [42,43]. The UHTC specimen [40] was heated vertically from the bottom through the hole on the workbench. The diameter of the heating spot was 20 mm and the distance from the laser heating-lens mounted at the bottom of the gantry to the specimen was 200 mm. The vertical displacement of the UHTC specimen was fixed by a horizontal vice along the two borders. The minimum force was adjusted to produce a friction force to balance the weight of the specimen. 

Corundum ceramic plates were placed on the contact area between the UHTC specimen and the horizontal vice to reduce heat transfer. In this situation, the thermal expansion of the UHTC in a vertical direction was restricted upon heating and a compression stress was then induced along this direction. The CCG sensor was attached to the surface of the UHTC along the horizontal direction to measure its thermo-mechanical response.

The CCG sensor used in this study was fabricated using Ge-doped silica fibres at the Institute of Photonics Technology (Jinan University, Guangzhou, China). A high-purity alumina adhesive (Rebond 989FS, Cotronics Corp., New York, NY, USA) was used to connect the CCG to the specimen. The size of the specimen was 40 mm × 45 mm × 5 mm. The UHTC sample used in this study was fabricated from commercial ZrB_2_ (Northwest Institute for Non-ferrous Metal Research, Xi’an, China) and SiC powders (Weifang Kaihua Micro-powder Co. Ltd., Weifang, China). The powder mixtures of ZrB_2_ with 15% v/v SiC were ball-milled in ethanol for eight hours with a hard milling tool and dried in a rotating evaporator. Milled powder was then hot-pressed uniaxially in a boron nitride coated graphite die at 1950 °C for 60 min under a vacuum and 30 MPa of pressure was applied, giving it good oxidative stability up to 2000 °C [44]. 

A high-power fibre-coupled laser (DILAS Diodenlaser GmbH, Mainz-Hechtsheim, Germany. Laser class: Compact-980.10-1500C, output power: 1500W, wavelength: 980 nm ± 10 nm@25 °C) was used to heat the specimen in this study. The temperature of the specimen was controlled by adjusting the laser heater output power according to the temperature response data. An optical fibre-grating demodulator (si425 Optical Sensing Interrogator, Micron Optics, Inc., Atlanta, GA, USA) was used to record reflected wavelength signals. A thermocouple was mounted on the adhesive layer to measure the temperature of the specimen and a multi-channel temperature signal acquisition instrument (LR8402-21, HIOKI, Nagano, Japan) was used to record temperature responses. The thermocouple signal was mainly used for temperature compensation, which is generally adopted to decouple CCGs signals [45].

## 3. Experimental Results and Analysis

### 3.1. Experimental Results

**Figure 2:**
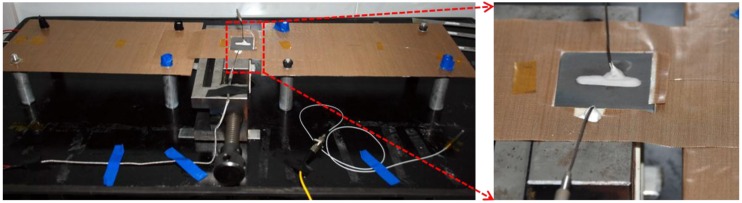
Experimental rig for the thermo-mechanical test.

The designed experimental rig is shown in Figure 2. The thermo-mechanical experimental test was performed using a CCG sensor with centre wavelengths of 1550.521 nm. During the test, the specimen was slowly heated by adjusting the output power of the laser heater, and the response of the CCG sensor and thermocouples was recorded by the instruments. The test stopped when the UHTC specimen fractured at 695 °C.

**Figure 3:**
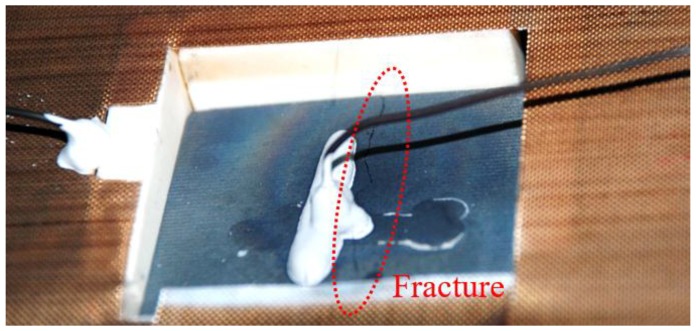
Photo of UHTC specimen with a fracture after a test.

The fracture was located at the centre of the specimen in an area parallel to the direction of the CCG sensor (Figure 3). During the test the specimen’s compression stress along the vertical direction increased alongside the temperature, because its expansion at that direction was constrained by the horizontal vice. Cracks were generated when local stress exceeded the UHTCs strength at that specific temperature, and the fracture occurred with propagating cracks due to the fragility of the UHTCs.

**Figure 4:**
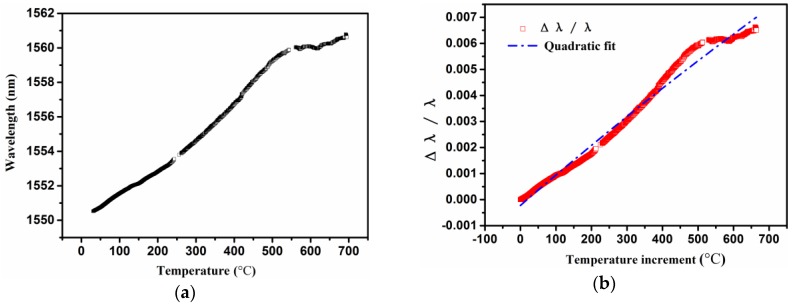
(**a**) Variation and (**b**) relative variation of wavelength alongside temperature.

The curve of the measured response signals from the CCGs versus the temperature is shown in Figure 4a. As can clearly be observed from that plot, the response of the wavelength presents a nonlinear increase versus the temperature. Nonlinearity is affected by many factors, such as the highly temperature-dependent nature of the thermo-optical coefficient and the thermal expansion coefficient of the silica fibres [46], the variation in the period of the optical fibre sensor and the elastic-optic coefficient [47]. Figure 4b shows the results of the relative variation in wavelength versus temperature, as well as the quadratic curve-fitting of the experimental data. Table 1 lists the curve fitting parameters with a 95% confidence interval. Similar quadratic thermal responses have been recorded in other studies [40,48,49,50].

### 3.2. Analysis of the Results

We have used Equation (1) [50] to denote the quadratic relationship between the relative variation of the measured total wavelength response $\Delta \lambda_{B}/\lambda_{B}$ and the temperature:

(1) $$\frac{\Delta \lambda_{B}}{\lambda_{B}}\;=\;K_{T1}\Delta T+K_{T2}\Delta T^{2}+C$$

where *K_T_*_1_ represents the coefficient of the first-order term, *K_T_*_2_ represents the coefficient of the second-order term, and *C* is a universal constant. The first-order term coefficient *K_T_*_1_ can also be written in the following form [40]:

(2) $$K_{T1}=\left(\alpha +\zeta \right)+K_{\varepsilon }\beta \left(\alpha_{s}-\alpha \right)$$

where *α*, *ζ* and *K_ε_* denote the thermal expansion coefficient, thermo-optical coefficient and strain sensitivity coefficient of the CCGs, respectively. The term *α_s_* denotes the thermal expansion coefficient of the UHTC and *β* is the strain transfer coefficient used to characterise the strain difference between the structural and the actual sensed strains induced by the adhesive layer. The sensed strain can be represented by the actual structural strain multiplied by factor *β* [50]:

(3) $$\varepsilon_{C}=\beta (\alpha_{s}-\alpha )\Delta T$$

where *ε_C_* denotes the CCGs-sensed strain along the horizontal direction for this test.

From Equations (2) and (3) it is possible to derive the following relation:

(4) $$\varepsilon_{C}=\frac{K_{T1}-\left(\alpha +\zeta \right)}{K_{\varepsilon }}\Delta T$$

The strain values *ε_C_* sensed by CCGs can be calculated using Equation (4) from the experimental data. Then, the practical structural strain can be calculated by the following equation, while the values of *β* at different temperatures can be found from [40]:

(5) $$\varepsilon_{S}=\varepsilon_{C}/\beta$$

**Figure 5:**
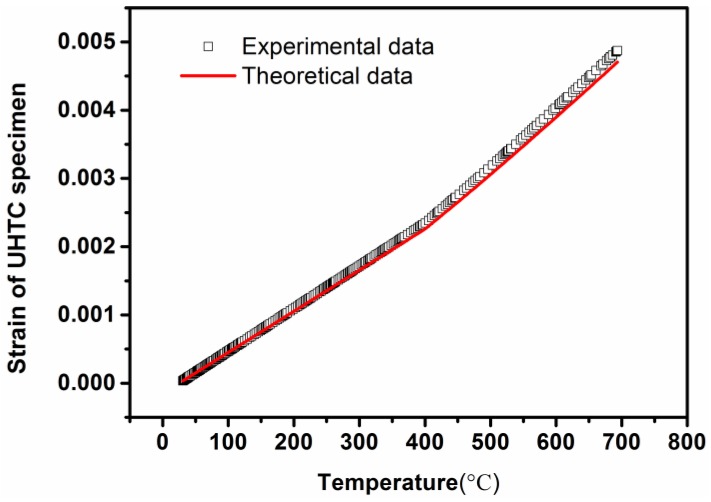
Comparison of theoretical and experimental strains.

The theoretical strain of the specimen can be solved by considering the thermal deformation relations. If we denote *ε_Th_* as the theoretical strain of the specimen along the horizontal direction (CCGs’ direction), its final value contains the effects of the strain in both horizontal and vertical directions:

(6) $$\varepsilon_{Th}=\left(\alpha_{s}-\alpha \right)\Delta T+\upsilon \left(\alpha_{s}-\alpha \right)\Delta T$$

where υ is the Poisson’s ratio of the UHTC material. Figure 5 shows a comparison between the experimental and theoretical strain. One can observe the existence of good agreement between the two data sets. The nonlinear experimental strain in Figure 5 was calculated by using Equation (4), in which the parameter *K_T_*_1_ has been obtained from the response signals of the CCGs. The theoretical strain in Figure 5 was calculated by using Equation (5). The maximum relative error is 2.15%, indicating that the CCGs sensor can provide a good strain measurement under thermo-mechanical loading for the external hot structure to which it is applied.

## 4. Finite Element Analysis and Discussions

### 4.1. Finite Element Model

**Figure 6:**
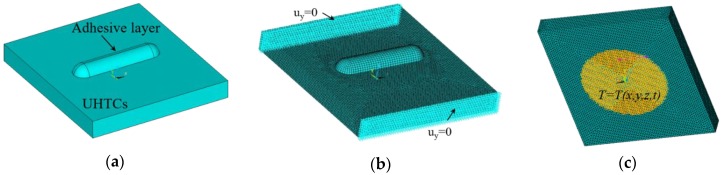
(**a**) Finite element model; (**b**) mechanical and (**c**) thermal boundary conditions of finite element analysis.

A finite element method (FEM) was used to simulate the thermo-mechanical test process. It is worth mentioning that the use of a robust FE model to represent the phenomenon could be instrumental to reduce the time available for the experimental tests, and especially the financial costs associated to full-scale experimental trials. A full-size three-dimensional model of the UHTC specimen and the adhesive layer was built using the finite element analysis (FEA) software (ANSYS, Version 16.1), as shown in Figure 6a. The FE model was assumed to be a combination of two quarter-spheres and a half-cylinder, approximately. The detailed dimensions of the adhesive layer and UHTC specimen used in this study are shown in Table 2. The CCG sensor was ignored in the FE model due to its small size. The material properties of the UHTC and adhesive are shown in Table 3, Table 4 and Table 5. The properties not listed in the tables at other temperatures can be estimated by linear interpolation. Heat transfer analyses were carried out with three dimensional twenty-node DC3D20 elements. Thermal-mechanical analyses were carried out with three dimensional twenty-node C3D20 elements. A mesh convergence study had been performed, and showed that the mesh size was sufficient for the stress within the critical region to converge.

**Figure 7:**
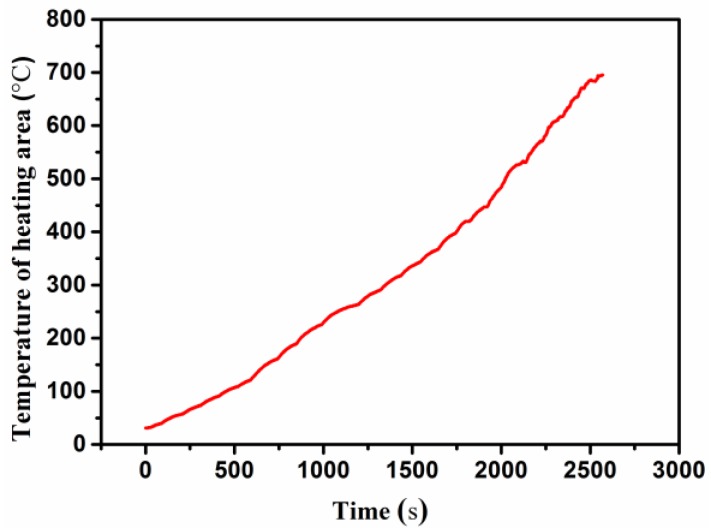
Temperature history curve of the experimental test.

The mechanical and thermal boundary conditions are illustrated in Figure 6b,c. The displacements of two side faces of the specimen along the vertical direction were fixed to the constraints representing the horizontal vice. The temperature history curve measured during the experimental test (Figure 7) was applied to the bottom surface of the specimen as the temperature boundary condition. The total heating time was 2570 s. Heating area was a 25 mm diameter circular area, which is the same as that of the laser heater spot. Other areas of the bottom and top surface were specified as the radiation boundary condition with a surface emissivity of 0.85. Natural convection was not considered in this study. Both the UHTC and adhesive layer were assumed be isotropic materials and remained in the linear elastic range during analysis, and they were always perfectly bonded to each other. The experimental simulation was carried out based on the sequential coupled thermo-mechanical analysis with a Newton-Raphson method.

### 4.2. Results and Discussion

**Figure 8:**
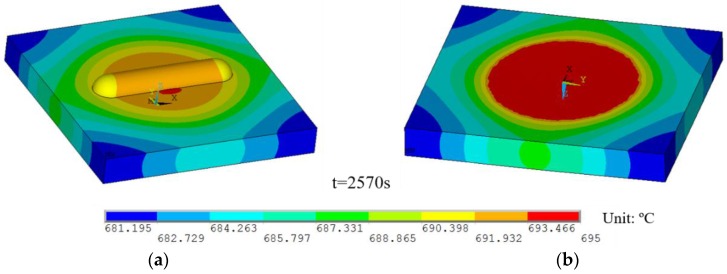
Temperature contour of (**a**) top view and (**b**) bottom view at 2570 s.

The temperature distributions at the end of the thermal analysis are given in Figure 8. It can be seen that the maximum temperature of the specimen at the centre reached the value of 695 °C while the minimum temperature was 681.2 °C and localised at the corners of the specimen. The large thermal conductivity value of the UHTCs material made the maximum temperature difference lower than 14 °C.

**Figure 9:**
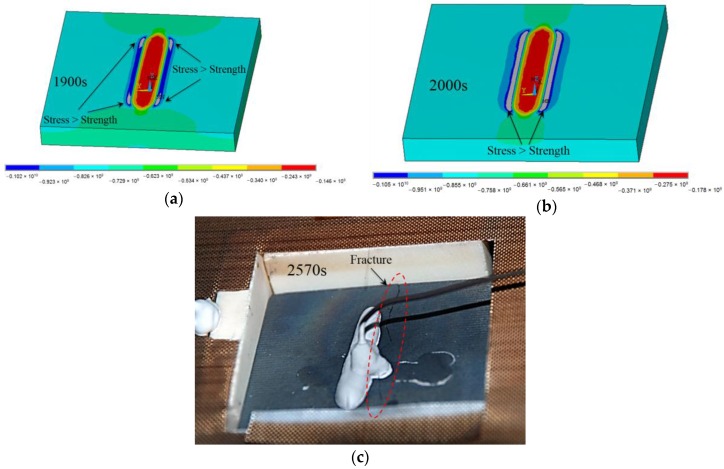
Comparison of failure positions between FEA and test at (**a**) 1900 s; (**b**) 2000 s; (**c**) 2570 s.

The temperature field distribution has been used as a thermal load to predict the internal stresses in the structure. The transient stress analysis results showed that the primary stress on the specimen was a compressive one (vertical to the CCG), and was caused by the constraints provided by the horizontal vice. By comparing the thermal stress contours at different times, it is possible to draw the same conclusion about the specimen’s stress along the y-direction: stresses are always negative (i.e., compressive), and the minimum compressive stress in absolute value is located at the bonding region between the UHTC and the adhesive layer, while the maximum compressive stress (magnitude) is located in the area around the boundary of the bonding area. The compressive stress distributions along the y-direction without an adhesive layer at 1900 s and 2000 s are shown in Figure 9a,b. The compressive stress at the four borders reached 1053 MPa at 1900 s (Figure 9a) and exceeded the UHTCs’ compressive tensile strength at that specific temperature (446 °C).

According to the FE analysis, the temperature of the border after 2000 s was around 484 °C, and the corresponding compressive strength was 1048 MPa. Two strip-shaped areas near the connection region exceeded their compressive strength (Figure 9b). If we compare the locations of the failure with the fracture point shown in Figure 9c, it is apparent that the fracture occurred in the location corresponding to the maximum compressive stress. The FEA results provide evidence to justify the observed experimental results. The micro-cracks present in the structure may have been generated as a result of the constrained thermal expansion after 2000 s, followed by crack propagation until fracture occurred.

**Figure 10:**
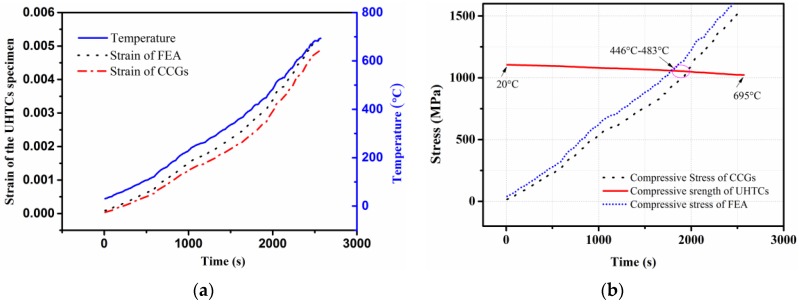
The curves of (**a**) strain and (**b**) stress over time.

The strain on the specimen can also be obtained from the FEA analysis. We selected all the nodes along the line where the CCG was bonded at the UHTCs’ adhesive surface, then calculated the average value of the nodal mechanical strains along the x-direction and obtained the strain versus time and temperature curves. These numerical values were then compared with the strain values measured by the CCG sensor (Figure 10a). By comparing the finite element simulation results with the experimental data we found that the results of the two methods are in good agreement. Table 6 provides the relative error of the two results, which decreased for increasing temperatures. The relative error was however larger in the low temperature regime (less than 100 °C), due to the strain value being itself very small. Above 200 °C the relative error was less than 15%, and the relative error between the two methods at the final temperature of 695 °C was only 6.71%.

Within the elastic range we can use the strain to calculate the corresponding stresses, according to Hooke’s law. As shown in Figure 10b, the structural compressive stress curves were calculated over time by using the strain of the FEA and the CCG sensor. At the same time, the compressive strength of the UHTC at the corresponding temperature was also obtained (see Figure 10b). Both compressive stresses were greater than the material compressive strength in the 1900 s–2000 s range, and the temperature range of the corresponding times were roughly 446 °C–483 °C, respectively. This was in agreement with the experimental results.

The discrepancies between simulations and experimental results were mainly derived from the representation of the boundary conditions and assumptions like the use of adiabatic boundary conditions. In the actual experiment, it was difficult to completely insulate the specimen from the surrounding environment and to keep the applied displacement constraints constants. Nonetheless, the results show that the FE model is capable to represent the physics and provide some robust predictions about the thermo-mechanical coupling occurring inside the structure.

## 5. Conclusions 

In this work, the application of a CCG fibre optic sensor on a hot structure was evaluated and a simple method to perform a combined thermal and mechanical load test has been presented. A high temperature test was performed on a UHTC specimen that was subjected to thermo-mechanical loads. The response characteristics of the bonded CCG were highlighted by a signal decoupling technique on the basis of a temperature compensation method. The results showed that the relative error between the test results and the theoretical strain value was less than 2.15%. The finite element method was used to simulate the experimental process, and the temperature field, stress field and strain field were calculated and analysed. The finite element analysis showed that the compressive stress of the connecting region exceeded the strength value of the material in the thermo-mechanical coupling environment, which explains the failure of the hot supporting structure. Numerical simulations can accurately predict the location and time of the failure of thermal structures, as well as the corresponding stress failure values. The numerical analysis can also reasonably explain the failure mechanism observed during the test. Furthermore, the numeric simulation results showed that the relative error of the strain decreased alongside an increase in temperature. The relative error was lower than 15% when the temperature was higher than 200 °C and only 6.71% at 695 °C. 

## Figures and Tables

**Table 1:** Fitting parameters of experimental data (R^2^ = 0.986).

Parameter	Value	Standard Error
Coefficient of the first-order term *K_T_*_1_	1.18 × 10^−5^	9.98 × 10^−8^
Coefficient of the second-order term *K_T_*_2_	−1.39 × 10^−9^	1.52 × 10^−10^
Universal constant C	−2.20 × 10^−4^	1.28 × 10^−5^

**Table 2:** Dimensions of the adhesive layer and UHTC specimen.

Parameter	Adhesive Layer			UHTC Specimen		
	Length	Width	Height (Sagitta)	Length	Width	Thickness
Value (mm)	20	6	2.5	45	40	5

**Table 3:** Mechanical properties of the UHTC material [51,52].

Density (kg/m^3^)	Poisson’s Ratio	Elastic Modulus (GPa)		Compressive Strength (MPa)	
		20 °C	1400 °C	20 °C	800 °C
4960	0.165	463.0	158.7	1106.4	1009.2

**Table 4:** Thermal properties of the UHTC material [40,53].

T (°C)	20	303	594	891	1196	1499	1806
Thermal conductivity (W/m·°C)	112.00	110.63	88.86	67.70	61.67	64.04	50.77
Specific heat (J/kg·°C)	700.00	777.61	828.19	869.36	1013.46	1016.08	1083.27
CTE (10^−6^ °C^−1^)	3.31	5.45	6.67	7.20	7.62	8.03	8.43

**Table 5:** Properties of the adhesive material [54].

Density (kg/m^3^)	CTE (10^−6^ °C^−1^)	Thermal Conductivity (W/m·°C)	Specific Heat (J/kg·°C)	Elastic Modulus (GPa)	Poisson’s Ratio
3700	8.1E-6	85.18	1004.16	370	0.2

**Table 6:** Relative errors of mechanical strain between FEA and experimental results.

Temperature (°C)	100	200	300	400	500	600	695
Relative Errors	18.96%	14.92%	13.79%	13.19%	9.91%	8.10%	6.71%

## References

[1] Mihailov S.J. (2012). Fiber bragg grating sensors for harsh environments. Sensors.

[2] Panopoulou A., Roulias D., Loutas T., Kostopoulos V. (2012). Health monitoring of aerospace structures using fibre bragg gratings combined with advanced signal processing and pattern recognition techniques. Strain.

[3] Su D., Qiao X., Yang H., Rong Q., Bai Z., Wang Y., Feng Z. (2014). Temperature-independent fiber inclinometer based on orthogonally polarized modes coupling using a polarization-maintaining fiber bragg grating. Sensors.

[4] Wang J.-N., Tang J.-L. (2010). Feasibility of fiber bragg grating and long-period fiber grating sensors under different environmental conditions. Sensors.

[5] Culshaw B. (2000). Measuring strain using optical fibres. Strain.

[6] Bennion I., Zhang L., Everall L. (2000). In-fibre grating techniques for strain sensing. Strain.

[7] Canning J., Stevenson M., Bandyopadhyay S., Cook K. (2008). Extreme silica optical fibre gratings. Sensors.

[8] Bueno A., Kinet D., Mégret P., Caucheteur C. (2013). Fast thermal regeneration of fiber bragg gratings. Opt. Lett..

[9] Fokine M. (2002). Thermal Stability of Chemical Composition Gratings in Fluorine-Germanium-Doped Silica Fibers. Opt. Lett..

[10] Fokine M. (2002). Photosensitivity, Chemical Composition Gratings and Optical Fiber Based Components. Ph.D. Thesis.

[11] Fokine M. (2002). Formation of thermally stable chemical composition gratings in optical fibers. J. Opt. Soc. Am. B.

[12] Pal S., Mandal J., Sun T., Grattan K., Fokine M., Carlsson F., Fonjallaz P.Y., Wade S., Collins S. (2003). Characteristics of potential fibre bragg grating sensor-based devices at elevated temperatures. Meas. Sci. Technol..

[13] Fokine M. (2004). Thermal stability of oxygen-modulated chemical-composition gratings in standard telecommunication fiber. Opt. Lett..

[14] Holmberg P., Laurell F., Fokine M. (2015). Influence of pre-annealing on the thermal regeneration of fiber bragg gratings in standard optical fibers. Opt. Express.

[15] Zhang B. (2007). Optical High Temperature Sensor Based on Fiber Bragg Grating. Ph.D. Thesis.

[16] Zhang B., Kahrizi M. (2007). High-temperature resistance fiber bragg grating temperature sensor fabrication. IEEE Sens. J..

[17] Bandyopadhyay S., Canning J., Stevenson M., Cook K. (2008). Ultrahigh-temperature regenerated gratings in boron-codoped germanosilicate optical fiber using 193 nm. Opt. Lett..

[18] Canning J., Stevenson M., Fenton J., Aslund M., Bandyopadhyay S. Strong regenerated gratings. Proceedings of the 20th International Conference on Optical Fibre Sensors.

[19] Canning J., Cook K., Aslund M., Stevenson M., Biswas P., Bandyopadhyay S. (2010). Regenerated Fibre Bragg Gratings.

[20] Barrera D., Finazzi V., Coviello G., Bueno A., Sales S., Pruneri V. Chemical composition gratings in germanium doped and boron-germanium co-doped fibers. Proceedings of the Optical Sensing and Detection.

[21] David B., Vittoria F., Joel V., Antonio B., Salvador S., Valerio P. (2010). On the Use of Optical Fiber Sensors (CCGs and PCFI) for Harsh Environments. Waves.

[22] Barrera D. (2010). Fiber-optic sensors for high-temperature applications. SPIE Newsroom.

[23] Li G., Guan B.O. Research on reflectivity of chemical composition grating sensors at high temperatures. Proceedings of the Passive Components and Fiber-Based Devices VII.

[24] Li G., Guan B.O. (2011). Improvement on reflectivity of chemical composition gratings at high temperatures. Microw. Opt. Technol. Lett..

[25] Li G., Liu M., Li Y., Guan B.O. (2012). Fabrication and sensing characteristics of the chemical composition grating sensor at high temperatures. Microw. Opt. Technol. Lett..

[26] Grobnic D., Smelser C.W., Mihailov S.J., Walker R.B. (2006). Long-term thermal stability tests at 1000 °C of silica fibre bragg gratings made with ultrafast laser radiation. Meas. Sci. Technol..

[27] Coviello G., Finazzi V., Villatoro J., Pruneri V. (2009). Thermally stabilized pcf-based sensor for temperature measurements up to 1000 °C. Opt. Express.

[28] Cheong Y., Chong W., Chong S., Lim K., Ahmad H. (2014). Regenerated type-iia fibre bragg grating from a Ge-B codoped fibre via thermal activation. Opt. Laser Technol..

[29] Laffont G., Cotillard R., Ferdinand P. (2013). Multiplexed regenerated fiber bragg gratings for high-temperature measurement. Meas. Sci. Technol..

[30] Lindner E., Canning J., Chojetzki C., Brückner S., Becker M., Rothhardt M., Bartelt H. (2011). Thermal regenerated type iia fiber bragg gratings for ultra-high temperature operation. Opt. Commun..

[31] Méndez A., Wnuk V.P., Fokine M., Claesson Å., Nilsson L.-E., Ferguson S., Graver T. Packaging process of fiber bragg grating strain sensors for use in high-temperature applications. Proceedings of the Fiber Optic Sensor Technology and Applications IV.

[32] Wnuk V.P., Méndez A., Ferguson S., Graver T. Process for mounting and packaging of fiber bragg grating strain sensors for use in harsh environment applications. Proceedings of the Smart Structures and Materials 2005: Smart Sensor Technology and Measurement System.

[33] Selfridge R.H., Schultz S.M., Lowder T.L., Wnuk V.P., Méndez A., Ferguson S., Graver T. Packaging of surface relief fiber bragg gratings for use as strain sensors at high temperature. Proceedings of the Smart Structures and Materials 2006: Smart Sensor Monitoring Systems and Applications.

[34] Reddy P.S., Sai Prasad R.L., Srimannarayana K., Shankar M.S., Gupta D.S. (2010). A novel method for high temperature measurements using fiber bragg grating sensor. Opt. Appl..

[35] Mamidi V.R., Kamineni S., Ravinuthala L.S.P., Thumu V., Pachava V.R. (2014). Characterization of encapsulating materials for fiber bragg grating-based temperature sensors. Fiber Integr. Opt..

[36] Reddy P.S., Prasad R.L.N.S., Gupta D.S., Shankar M.S., Narayana K.S., Kishore P. (2011). Encapsulated fiber bragg grating sensor for high temperature measurements. Opt. Eng..

[37] Barrera D., Finazzi V., Villatoro J., Sales S., Pruneri V. Performance of a high-temperature sensor based on regenerated fiber bragg gratings. Proceedings of the 21st International Conference on Optical Fibre Sensors (OFS21).

[38] Barrera D., Finazzi V., Villatoro J., Sales S., Pruneri V. (2012). Packaged optical sensors based on regenerated fiber bragg gratings for high temperature applications. IEEE Sens. J..

[39] Azhari A., Liang R., Toyserkani E. (2014). A novel fibre bragg grating sensor packaging design for ultra-high temperature sensing in harsh environments. Meas. Sci. Technol..

[40] Xie W., Meng S., Jin H., Du C., Wang L., Peng T., Scarpa F., Huo S. (2016). Measurement of the high-temperature strain of uhtc materials using chemical composition gratings. Meas. Sci. Technol..

[41] Glass D.E. Ceramic matrix composite (CMC) thermal protection systems (TPS) and hot structures for hypersonic vehicles. Proceedings of the 15th AIAA Space Planes and Hypersonic Systems and Technologies Conference.

[42] Latini V., Striano V., Coppola G., Rendina I. Fiber optic sensors system for high-temperature monitoring of aerospace structures. Proceedings of the Photonic Materials, Devices, and Applications II.

[43] Manor D., Lau K.Y., Johnson D.B. (2005). Aerothermodynamic environments and thermal protection for a wave-rider second stage. J. Spacecr. Rockets.

[44] Zhang X.-H., Hu P., Han J.-C. (2008). Structure evolution of ZrB_2_-SiC during the oxidation in air. J. Mater. Res..

[45] Ge Y., Elshafie M.Z., Dirar S., Middleton C.R. (2014). The response of embedded strain sensors in concrete beams subjected to thermal loading. Constr. Build. Mater..

[46] Li G.-Y., Guan B.O. (2009). The strain response of chemical composition gratings at high temperatures. Meas. Sci. Technol..

[47] Maier R.R., MacPherson W.N., Barton J.S., Jones J.D., McCulloch S., Burnell G. (2004). Temperature dependence of the stress response of fibre bragg gratings. Meas. Sci. Technol..

[48] Meng S., Du C., Xie W., Huo S., Jiao L., Jin H., Song L. (2013). Application of high-temperature optical fiber sensor in temperature and strain testing of hot structure. J. Sol. Rocket Technol..

[49] Du C., Xie W., Meng S., Yin Y., Jiao L., Song L. The connection technology based on high temperature silica fiber optic sensor. Proceedings of the Sensors and Smart Structures Technologies for Civil, Mechanical, and Aerospace Systems 2012.

[50] Du C., Xie W., Huo S., Meng S., Xu K., Jiao L. The response of high-temperature optical fiber sensor applied to different materials. Proceedings of the Fourth International Conference on Smart Materials and Nanotechnology in Engineering.

[51] Wang L., Fang G., Liang J., Wang C. (2014). Formation mechanism and high temperature mechanical property characterization of SiC depletion layer in ZrB_2_/SiC ceramics. Mater. Charact..

[52] Wang L.-L., Liang J., Fang G.-D., Wan X.-Y., Xie J.-B. (2014). Effects of strain rate and temperature on compressive strength and fragment size of ZrB_2_-SiC-Graphite composites. Ceram. Int..

[53] Wang Z., Hong C., Zhang X., Sun X., Han J. (2009). Microstructure and thermal shock behavior of ZrB_2_-SiC-Graphite composite. Mater. Chem. Phys..

[54] 3000 °F Resbond^TM^ 989FS High Purity Alumina Adhesive Rapid Curing Formulation. http://www.cotronics.com/WEB%20SHEETS/989FS%20NP.pdf.

